# Preoperative MRI Findings Predict Two-Year Postoperative Clinical Outcome in Lumbar Spinal Stenosis

**DOI:** 10.1371/journal.pone.0106404

**Published:** 2014-09-17

**Authors:** Pekka Kuittinen, Petri Sipola, Ville Leinonen, Tapani Saari, Sanna Sinikallio, Sakari Savolainen, Heikki Kröger, Veli Turunen, Olavi Airaksinen, Timo Aalto

**Affiliations:** 1 Department of Neurosurgery, Kuopio University Hospital, Kuopio, Finland and Institute of Clinical Medicine, University of Eastern Finland, Kuopio, Finland; 2 Department of Clinical Radiology, Kuopio University Hospital, Kuopio, Finland and Unit of Radiology, Institute of Clinical Medicine, University of Eastern Finland, Kuopio, Finland; 3 Department of Neurosurgery, Kuopio University Hospital, Kuopio, Finland and Unit of Neurosurgery, Institute of Clinical Medicine, University of Eastern Finland, Kuopio, Finland; 4 Department of Clinical Radiology, Kuopio University Hospital, Kuopio, Finland; 5 Institute of Public Health and Clinical Nutrition, University of Eastern Finland, Kuopio, Finland; 6 Department of Neurosurgery, Kuopio University Hospital, Kuopio, Finland; 7 Department of Orthopaedics and Traumatology, Kuopio University Hospital and Bone and Cartilage Research Unit, University of Eastern Finland, Kuopio, Finland; 8 Department of Orthopaedics and Traumatology, Kuopio University Hospital, Kuopio, Finland; 9 Department of Physical and Rehabilitation Medicine, Kuopio University Hospital, Kuopio, Finland; 10 Health Center Ikioma OY, Mikkeli, Finland; University of Kansas, United States of America

## Abstract

**Purpose:**

To study the predictive value of preoperative magnetic resonance imaging (MRI) findings for the two-year postoperative clinical outcome in lumbar spinal stenosis (LSS).

**Methods:**

84 patients (mean age 63±11 years, male 43%) with symptoms severe enough to indicate LSS surgery were included in this prospective observational single-center study. Preoperative MRI of the lumbar spine was performed with a 1.5-T unit. The imaging protocol conformed to the requirements of the American College of Radiology for the performance of MRI of the adult spine. Visual and quantitative assessment of MRI was performed by one experienced neuroradiologist. At the two-year postoperative follow-up, functional ability was assessed with the Oswestry Disability Index (ODI 0–100%) and treadmill test (0–1000 m), pain symptoms with the overall Visual Analogue Scale (VAS 0–100 mm), and specific low back pain (LBP) and specific leg pain (LP) separately with a numeric rating scale from 0–10 (NRS-11). Satisfaction with the surgical outcome was also assessed.

**Results:**

Preoperative severe central stenosis predicted postoperatively lower LP, LBP, and VAS when compared in patients with moderate central stenosis (p<0.05). Moreover, severe stenosis predicted higher postoperative satisfaction (p = 0.029). Preoperative scoliosis predicted an impaired outcome in the ODI (p = 0.031) and lowered the walking distance in the treadmill test (p = 0.001). The preoperative finding of only one stenotic level in visual assessment predicted less postoperative LBP when compared with patients having 2 or more stenotic levels (p = 0.026). No significant differences were detected between quantitative measurements and the patient outcome.

**Conclusions:**

Routine preoperative lumbar spine MRI can predict the patient outcome in a two-year follow up in patients with LSS surgery. Severe central stenosis and one-level central stenosis are predictors of good outcome. Preoperative finding of scoliosis may indicate worse functional ability.

## Introduction

Lumbar spinal stenosis (LSS) is defined as “buttock or lower extremity pain, which may occur with or without low back pain (LBP), associated with diminished space available for the neural and vascular elements in the lumbar spine” [1], [2]. LSS is the most common indication for lumbar spinal surgery in people aged over 65 years. Incidence of lumbar spinal stenosis is increasing due to the aging population, which increase also the frequency of more complex lumbar spine procedures, which in turn is associated with the more demand for the healthcare [3]. The aim of surgery is to improve functional ability and relieve symptoms with adequate decompression of the neural elements. However, the long-term results of surgery are good to excellent only in two-thirds of patients [3], [4]. Accordingly, preoperative patient selection is considered critical [5]–[8]. Clinically, routine magnetic resonance imaging (MRI) is the standard method in the diagnostic workup of patients with suspected LSS [9]–[10]. However, impacts of the MRI findings to the patients’ symptoms have been also questioned [11].

We have earlier reported that depressive symptoms are a strong predictor for a worse short-term outcome [12], [13] and for the two-year outcome in LSS surgery [7]. Depression and disability were also clearly associated in a cross-sectional setting [14].

There are several a cross-sectional studies on preoperative radiological findings and preoperative patient’s symptoms, but only few with prospective setting. A clear association in a cross-sectional setting has been reported between the minimum dural sac cross-sectional (DSCA) area in lumbar MRI and several outcome measures (walking ability, symptom severity, quality of life) with the 82 and 88 LSS patient groups [15], [16]. In another study with 50 patients population a smaller central anterior–posterior (AP) canal have reported greater perceived disability, but no other group differences emerged [17]. In contrast, a lack of association has been reported between the ODI and DSCA, qualitative evaluation of the lateral recess, and foraminal stenosis with the 63 LSS patients [18]. Thus there is discrepancy in the previous literature.

Yukawa et al reported in their prospective study with the 62 LSS patients that multilevel central stenosis were, on average, older and walked a shorter distance preoperatively and postoperatively, although the improvement in their postoperative self-assessment scores was similar to that of patients with single-level stenosis [19]. Sigmundsson et al. investigated the predictive value of MRI findings among a study population consisting of 109 LSS patients undergoing surgery with a one-year follow-up. They found in their prospective study that a smaller dural sac area predicted less leg pain postoperatively and more pain relief in low LBP [20]. None of these studies have, however, investigated the predictive value of visual and quantitative findings from preoperative lumbar spine MR images for both subjective and objective clinical outcome measures with a two-year follow-up [19], [20].

The use of the standardized Oswestry Disability Index (ODI) [21], [22], visual analogue scale for pain (VAS) [23], Beck Depression Inventory (BDI) [24], and specific back pain and leg pain assessment with a numeric rating scale (NRS-11) [25] has improved the accuracy and reproducibility in reliably grading functional disability, pain and depressive symptoms in patients. Keeping in mind the strong association of depressive symptoms and outcome measures of LSS, depressive symptoms should be adjusted. As far as we are aware, there have been no earlier LSS studies on MRI predictors that have adjusted the clinical outcome for depressive symptoms.

The purpose of the current study was to investigate the predictive value of preoperative MRI findings for the postoperative clinical outcome by comparing the preoperative imaging findings with the postoperative symptoms and function measured using standardized methods in a prospective study setting in LSS.

## Materials and Methods

### Patients

This prospective single-center study was approved by the Ethics Committee of Kuopio University Hospital, and the patients provided written informed consent to participate this study which was also documented. Ethics committee approved this procedure. The original study population consisted of 102 LSS patients, including 84 patients with central stenosis and lateral stenosis, and 18 patients having only lateral stenosis (5, 7, 13, 26). In the current study we included only these 84 central stenosis patients (mean age 63±11 years, male 43%) with both clinically and radiologically defined LSS who had been selected for surgical treatment. Selection for surgery was made by an orthopedist or neurosurgeon at Kuopio University Hospital, Kuopio, Finland. The inclusion criteria were: 1) the presence of severe back, buttock, and/or lower extremity pain, with radiographic evidence (computed tomography, magnetic resonance imaging, myelography) of compression of the cauda equina or exiting nerve roots by degenerative changes (ligamentum flavum, facet joints, osteophytes, and/or disc material), and 2) the surgeon’s judgment in clinical and radiological evaluation that the patient had degenerative LSS requiring operative treatment. In addition, all patients had a history of ineffective response to conservative treatment.

The exclusion criteria for this current study were: pure lateral stenosis; emergency or urgent spinal surgery precluding recruitment and protocol investigations; cognitive impairment prohibiting completion of the questionnaires or other failures in co-operation, and the presence of metallic particles in the body preventing the magnetic resonance imaging investigation. A previous spine operation or coexisting disc herniation were not exclusion criteria, but the main diagnosis of the study patients had to be LSS. The surgeons sent the information on eligible patients to the Department of Physical and Rehabilitation Medicine, which organized the study [26].

MRI was performed preoperatively for all patients, and functional ability, clinical symptoms, and patient satisfaction were assessed at the two-year follow-up.

### Magnetic resonance imaging

MR imaging of the lumbar spine was performed with a 1.5-T imager (Vision; Siemens Medical Solutions, Erlangen, Germany) and a dedicated receive-only spine coil. All patients were imaged prospectively with the same study protocol for study purposes. The imaging protocol conformed to the requirements of the American College of Radiology for the performance of MRI of the adult spine [27]. The following sequences were used: (a) sagittal T1-weighted spin-echo (repetition time/echo time (TR/TE) 600/12 ms; flip angle, 150°; 4-mm sections; intersection gap, 0.4 mm; field of view (FOV), 290 mm; rectangular FOV, 80%; three signals acquired per data line; matrix 288×512); (b) sagittal T2-weighted fast spin-echo (3500/120; flip angle, 180°; echo train length of five; 4-mm sections; intersection gap, 0.4 mm; FOV 290 mm; rectangular FOV, 63%; two signals acquired; matrix 180×512); (c) transverse T1-weighted spin-echo (700/15; flip angle, 90°; 4-mm sections; intersection gap, 0.4 mm; FOV, 250 mm; rectangular FOV, 80%; two signals acquired per data line; matrix 288×512); and (d) transverse T2-weighted fast spin-echo (5000/120; flip angle, 180°; echo train length of 15; 4-mm sections; intersection gap, 0.4 mm; FOV, 250 mm; rectangular FOV, 100%; three signals acquired per data line; matrix 330×512).

The entire lumbar spine was studied from the sagittal images (T12-S1), including parasagittal imaging of all the neural foramina bilaterally. Transverse images were obtained from the inferior aspect of L1 to the inferior aspect of S1, and the orientation of the sections was planned parallel to the major axis of each disc. In all sequences, a saturation band was placed over abdominal vessels.

### MRI predictors

Image evaluation was performed with Numaris software (Siemens Medical Systems) by a neuroradiologist with 15 years of experience of spinal MRI (T.S.). Image analysis was performed independently without knowledge of the patients’ clinical symptoms and data. Each level from the inferior aspect of L1 to the inferior aspect of S1 was analyzed separately. The central spinal canal was evaluated both visually and quantitatively. The lateral recess, lateral foramen, scoliosis, stenotic levels and spondylolisthesis were evaluated visually. The central canal was visually classified into three grades: 0 = normal or mild changes (ligamentum flavum hypertrophy and/or osteophytes and/or or disk bulging without narrowing in the central spinal canal); 1 = moderate stenosis (central spinal canal is narrowed but spinal fluid is still clearly visible between the nerve roots in the dural sac); 2 = severe stenosis (central spinal canal is narrowed and there is only a faint amount of spinal fluid or no fluid between the nerve roots in the dural sac). In quantitative image evaluation, each level was first assessed visually. On the image with the visually smallest cross-sectional area of the dural sac (mm^2^), this area was manually traced. The number of stenotic levels was graded as: 1 = 1 stenotic level, 2 = two stenotic levels, 3 = three stenotic levels, 4 = four stenotic levels. The number of stenotic levels was also dichotomously classified as 1 (one-level stenosis) or 2 (two or more stenotic levels).

The lateral canal of the lumbar spine was divided into subarticular (entrance) and foraminal (mid) zones. The subarticular zone (lateral recess) was the most cephalad part of the lateral lumbar canal and located medial to or underneath the superior articular process. The foraminal zone was located below the pedicle. Each subarticular zone and foraminal zone was evaluated separately and bilaterally. In visual analysis, the grading system classified the lumbar nerve root canals into three grades: 0 = normal, 1 = narrowing without root compression and 2 = nerve root compression [28].

Scoliosis was evaluated visually and categorized into: 0 = no scoliosis, 1 = mild scoliosis, 2 = severe scoliosis. Spondylolisthesis was visually analyzed and categorized as 0 = no spondylolisthesis or 1 = spondylolisthesis.

### Assessment of postoperative symptoms, functional disability and satisfaction with surgical outcome

The overall current low back and leg pain intensity was assessed using a self-administered VAS (range 0–100 mm) in a sitting position during study visits. This has been demonstrated to be a valid index of experimental, clinical, and chronic pain [23].

Back pain at rest (during last week) and leg pain on walking (during last week) were measured separately with a numerical rating scale from 0–10 (NRS-11) [25]. The questions about pain were anchored on the left (0) with the descriptor “no pain” and on the right (10) with the descriptor “intolerable pain”.

Subjective disability was measured using the validated Finnish version of the ODI, where 0% represents no disability and 100% extreme debilitating disability [21]–[22], [29].

The treadmill test (0–1000 m) was supervised by a physiotherapist. The patient was asked to keep a straight upright position during walking (on a zero-degree ramp). The starting speed was 0.67 m/s for the first 10 min (400 m), then 1 m/s for the next 10 min (600 m), and the maximum result was thus 1000 m in 20 min. If the patient was unable to start with a speed of 0.67 m/s, another test with a starting speed of 0.5 m/s was applied.

Satisfaction with the surgical outcome was assessed using a seven-category scale as follows: −3 = surgery was a total failure; −2 = condition is now considerably worse; −1 = condition is now slightly worse; 0 = no change; 1 = condition has slightly improved; 2 = condition has considerably improved; and 3 = totally cured. With respect to satisfaction, a “good outcome” consisted of those patients who were either “totally cured” or reported “condition considerably improved”, whereas a “worse outcome” consisted of the other responses [26].

### Statistical analyses

Analysis was performed using a general linear univariate model, and for patient satisfaction using a generalized linear model. Adjusting factors in the analysis were the age at operation (years), spondylodesis (yes/no) at operation (with or without instrumentation), and depressive symptoms (Beck Depression Inventory as a continuous scale, 0–63) [24] at two-year follow-up.

The predictive value of the radiological factors was assessed as follows: all the MRI predictors and adjusting factors were included together in the model, and tested together against each outcome measure. We applied a backward stepwise method in the analysis, using SPSS for Windows (version 19.0; SPSS, IBM, Chicago IL, USA). Statistical significance was set at p<0.05.

## Results

### Preoperative clinical characteristics and surgical outcome

Patient characteristics are summarized in Table 1. The mean age of the study patients (n = 84) at the time of surgery was 63 years (range 33–83), and 36 (43%) of the subjects were male. Twelve patients (14%) had undergone a previous spine operation.

**Table 1:** Clinical characteristics of the study subjects (n = 84).

Phase	Preoperative phase	2-year post-operative	P-value
**Male/Female**	36/48 (43/57)		
**Marital status married or co-habiting**	54 (64)		
**Current smoker**	17 (20)		
**Previous lumbar operation**	12 (14)		
**Age**	63 (11.0)		
**BMI (kg/m^2^)**	29.6 (4.0)		
**Number of somatic diseases**	5.2 (3.0)		
**BDI score**	10.4 (6.1)	7.6 (5.7)	<0.001
**ODI**	44.8 (16.1)	27.1 (19.8)	<0.001
**VAS overall**	32.8 (24.5)	12.6 (17.7)	<0.001
**NRS LBP**	4.1 (2.6)	1.9 (2.3)	<0.001
**NRS LP on walking**	6.4 (2.6)	3.2 (2.7)	<0.001
**Walking distance (m)**	551 (446)	729 (384)	<0.001

Note: Except where indicated, the data are numbers of patients, with percentages or means ± standard deviations in parentheses.

P-values: Paired T-test was used.

ODI = Oswestry Disability Index scale (0–100), VAS overall = Visual analogue pain scale (0–100), VAS LBP = specific back pain scale (0–10), VAS LP = specific leg pain scale (0–10), BDI = Beck Depression Inventory (0–63).

All the pre- and postoperatively evaluated outcome measures displayed a statistically significant improvement after surgery (p<0.001; Table 1). Postoperative satisfaction was as follows: 9 (11.1%) “totally cured”; 39 (48.1%) “considerable improvement”; 25 (30.9%) “slight improvement”; 2 (2.5%) “no change”; 3 (3.7%) “condition is now slightly worse”; and 3 (3.7%) patients “considerably worse outcome”.

### Radiological findings

In visual analysis, none of the patients had a normal central canal. The central canal was moderately and severely stenosed in 40 (47.6%) and 44 (52.4%) patients, respectively. In quantitative analysis, the mean minimal DSCA was 55.2±20.9 mm2 (range 12–120). The lateral spinal canal recess was moderately and severely stenosed in 60 (71.1%) and 24 (28.9%) patients, respectively. The lateral spinal foramina was normal, moderately, and severely stenosed in 47 (55.4%), 30 (36.1%), and 7 (8.4%) patients, respectively. One-, two-, three-, and four-level central stenosis was observed in 29 (34.5%), 34 (40.5%), 13 (15.5%), and 8 (9.5%) patients, respectively. In dichotomous classification, one-level stenosis was recorded in 29 (34.5%) and stenosis of two or more levels in 55 (65.5%) patients. Scoliosis was severe in 3 (3.6%), mild in 19 (22.9%) and normal in 61 (73.5%) patients. One-level spondylolisthesis was found in 2 (2.4%) patients.

### Predictive value of imaging findings for 2-year postoperative outcome

In parentheses below, the means and standard deviations of the study groups are presented, in addition to p-values and statistical tests of subgroups not mentioned in the methods.

Severe stenosis predicted less postoperative LP compared to moderate stenosis (2.75±2.6 vs 4.25±3.1; p = 0.028). Nevertheless, the improvement in LP was statistically also significant among patients in the moderate stenosis group (p<0.001; paired t-test).

Similarly, severe stenosis predicted less postoperative LBP compared to moderate stenosis (1.6±2.3 vs 2.4±2.5; p = 0.046). The improvement in LBP was also statistically significant among patients in the moderate stenosis group (p<0.001; paired t-test).

**Figure 1:**
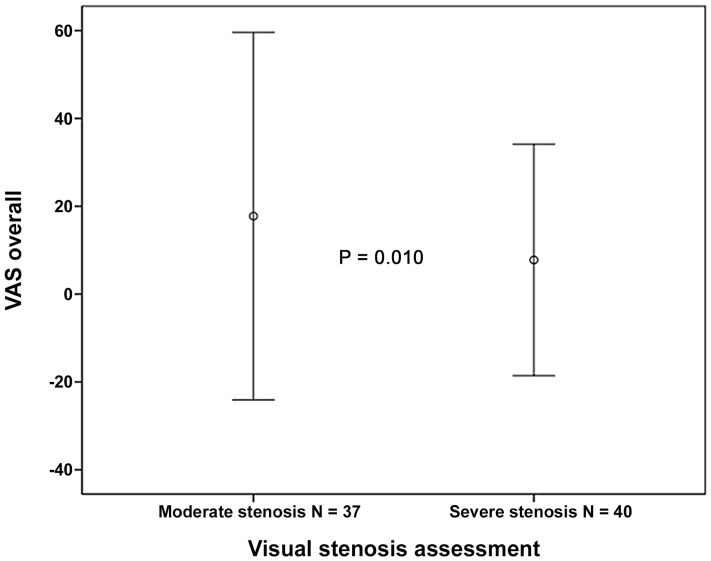
Visual analogue pain (mean ± SD) on two-year follow-up in patients with moderate and severe central spinal stenosis in visual analysis of lumbar spine magnetic resonance images.

Moreover, severe stenosis predicted a lower postoperative overall VAS score compared to moderate stenosis (7.8±13.2 vs 17.8±20.9; p = 0.010) (Figure 1). The improvement in the VAS score was also statistically significant among patients with moderate stenosis (p<0.001; paired t-test).

Finally, severe stenosis predicted better postoperative satisfaction with the surgical outcome compared to moderate stenosis (OR 0.297; 95% CI 0.100–0.880; p = 0.029).

Mild scoliosis predicted a worse 2-year outcome with the ODI compared to patients who had no scoliosis (34.3±21.5 vs 24.6±18.5; p = 0.031). The improvement in the ODI was also statistically significant among patients with scoliosis (p = 0.003; paired t-test).

In addition, scoliosis predicted a shorter postoperative treadmill test result compared to patients who had no preoperative scoliosis (547 m ± 464 m vs 820 m ± 315 m; p = 0.001). The improvement in walking ability in the treadmill test was not statistically significant among patients with scoliosis (p = 0.397; paired t-test).

One-level central stenosis predicted lower postoperative LBP compared to patients who had two or more stenotic levels (1.55±2.1 vs 2.22±2.5; p = 0.026).

We did not find any predictive value for quantitative evaluation of the central spinal canal or visual evaluation of spondylolisthesis, the lateral spinal canal recess and foramina.

## Discussion

Our main finding was that the visually evaluated severity of lumbar spinal stenosis correlated with the postoperative clinical outcome. Interestingly, in the visual classification of the central spinal canal, the LP, LBP, and overall VAS were postoperatively higher in patients with moderate than with severe central canal stenosis. In addition, more severe stenosis also associated with better postoperative satisfaction with the surgical outcome. However, according to subgroup analysis, patients with only moderate stenosis also displayed a statistically significant improvement in LP, LBP, and overall VAS. Thus, patients with only moderate stenosis still appear to experience significant pain relief following surgical treatment for LSS.

Mild scoliosis predicted a worse postoperative ODI and walking distance in the treadmill test compared with patients who had no scoliosis. However, despite the scoliosis, subgroup analysis revealed that patients had a significant improvement in the ODI but not in the walking distance in the treadmill test. Consistently with this, Frazier et al. observed that greater preoperative scoliosis predicted more postoperative back pain. However, their radiological evaluation was based on plain X-ray images [30]. In our study, scoliosis also predicted a worse postoperative outcome in the ODI and treadmill test, but not worse LBP. Thus, patients who have scoliosis still benefit from surgical treatment for LSS in terms of their overall functional ability, but the effect on walking ability appears to be non-significant.

Patients who preoperatively had only one stenotic level reported lower postoperative LBP than patients who had two or more stenotic levels. This could be expected, since the degenerative changes are then also often more severe. In contrast, Sigmundsson et al. found that multilevel stenosis patients had less leg pain postoperatively than patients with single-level stenosis [20]. Amundsen et al. did not find any association between the number of stenotic levels and the surgical outcome in their study [31].

In the literature, there are only a few earlier prospective studies on the predictive value of preoperative MRI findings for an adequately determined postoperative clinical outcome on two-year follow-up. Yukawa et al. observed a correlation between better postoperative ODI scores in patients who had a DSCA under 70 mm2 in preoperative MRI [19]. However, the authors did not visually evaluate the severity of stenosis, which we found an elementary part of image analysis, especially in patients with stenosis in the upper part of the lumbar spine. Sigmundsson et al. found in their prospective study that a smaller dural sac area predicted less leg pain postoperatively and more pain relief for LBP. However, they did not visually evaluate the severity of LSS, and walking distance was only subjectively estimated by the patient, depressive symptoms were not adjusted, and the clinical outcome was only evaluated with a one-year follow-up [20]. Our results are generally in line with these studies, i.e. more severe visually determined preoperative central canal stenosis predicted less pain and better satisfaction postoperatively.

Studies on visually analyzed spinal canal stenosis of the whole lumbar spine are rare. In our study, we found a clear correlation between visually assessed central spinal canal stenosis and the patient outcome, but no correlation in quantitative preoperative measurements. How can this discrepancy be explained? The amount of neural tissue at the L1–2 and L2–3 levels is significantly greater than at the L4–5 or presacral levels. Thus by measuring only the cross-sectional area of the dural sac, subjects with reduced space for neural tissue may not be correctly recognized. According to our findings, quantitative evaluation with the used methods cannot replace visual interpretation performed by an experienced radiologist. To the best of our knowledge, there have been no previous prospective studies in which the predictive value of lateral spinal stenosis has been examined. Despite the visually evaluated lateral spinal recess and foraminal stenosis not predicting any postoperative outcome in our study, it may have clinical relevance. Lateral stenosis, if not decompressed properly, might be associated with a poor outcome. All our patients had central canal stenosis, which is always associated with a stenotic lateral recess but only few had foraminal stenosis, which may explain our results.

The strengths of this study are the prospective, observational study setting, carefully characterized study population. The study included clinically relevant subjective and validated outcome measures together with objectively measured walking distance, and the analyses were adjusted for depressive symptoms, age, and fusion. A two-year follow-up is considered as a “golden standard” in spine surgery studies. The standardized MRI protocol was planned and carefully performed for the study purposes, and the evaluation was performed with visually and quantitatively by an experienced neuroradiologist.

The limitation of this study are relatively small number of the patients, however number of the patients in the previous prospective studies are less than in this study expect in the study by Sigmundsson et al where was several shortages compared to this study as pointed out earlier (20). In our study number of patients was sufficient for detecting clinically relevant associations.

The results of the current study relate to routine clinical MRI with patients lying in the supine position. Imaging patients in the supine position is also a limitation, because the symptoms may worsen in the upright position, and the upright position may also alter the anatomy of the neural canal. Accordingly, an upright position would be the most appropriate image acquisition position to link image findings to the patient’s symptoms [32], [33]. Hiwatashi et al. found in their study that axial loading with imaging can even influence treatment decisions [34].

The incidence of lumbar spinal stenosis is increasing due to the aging of population [35]. This also increase the number of LSS operations. However, the selection of patients for surgical treatment still remains challenging. Our results strengthen the classical conception that the diagnosis of this syndrome depends on the clinical history and radiographic evidence of a demonstrable stenosis [36], [37]. This study shows that pre-operative lumbar spine MRI imaging can predict the two-year clinical outcome in LSS surgery patients. The results of our study can be used to improve patient information and selection of patients for surgery.

## Conclusions

Routine preoperative lumbar spine MRI can predict the two-year clinical outcome in LSS surgery. Severe central stenosis, compared with moderate stenosis, predicted better postoperative satisfaction and less pain. One-level stenosis, compared to patients who had two or more stenotic levels, predicted less low back pain. Preoperative scoliosis may indicate a worse functional outcome.
